# Rapid Evolution of Enhanced Zika Virus Virulence during Direct Vertebrate Transmission Chains

**DOI:** 10.1128/JVI.02218-20

**Published:** 2021-03-25

**Authors:** Kasen K. Riemersma, Anna S. Jaeger, Chelsea M. Crooks, Katarina M. Braun, James Weger-Lucarelli, Gregory D. Ebel, Thomas C. Friedrich, Matthew T. Aliota

**Affiliations:** a University of Wisconsin–Madison, Madison, Wisconsin, USA; b University of Minnesota, Twin Cities, St. Paul, Minnesota, USA; c Virginia Tech, Blacksburg, Virginia, USA; d Colorado State University, Fort Collins, Colorado, USA; Cornell University

**Keywords:** *Aedes aegypti*, flavivirus, Zika virus, experimental evolution, host cycling, pathogenesis, virulence

## Abstract

Zika virus (ZIKV) has the unusual capacity to circumvent natural alternating mosquito-human transmission and be directly transmitted human to human via sexual and vertical routes. The impact of direct transmission on ZIKV evolution and adaptation to vertebrate hosts is unknown. Here, we show that molecularly barcoded ZIKV rapidly adapted to a mammalian host during direct transmission chains in mice, coincident with the emergence of an amino acid substitution previously shown to enhance virulence. In contrast, little to no adaptation of ZIKV to mice was observed following chains of direct transmission in mosquitoes or alternating host transmission. Detailed genetic analyses revealed that ZIKV evolution in mice was generally more convergent and subjected to more relaxed purifying selection than that in mosquitoes or alternate passages. These findings suggest that prevention of direct human transmission chains is paramount to resist gains in ZIKV virulence.

**IMPORTANCE** We used experimental evolution to model chains of direct and indirect Zika virus (ZIKV) transmission by serially passaging a synthetic swarm of molecularly barcoded ZIKV within and between mosquitoes and mice. We observed that direct mouse transmission chains facilitated a rapid increase in ZIKV replication and enhanced virulence in mice. These findings demonstrate that ZIKV is capable of rapid adaptation to a vertebrate host and indicate that direct human-to-human transmission poses a greater threat to public health than currently realized.

## Abstract (precis)

We used experimental evolution to model chains of direct and indirect Zika virus (ZIKV) transmission by serially passaging a synthetic swarm of molecularly barcoded ZIKV within and between mosquitoes and mice. We observed that direct mouse transmission chains facilitated a rapid increase in ZIKV replication and enhanced virulence in mice.

## INTRODUCTION

Zika virus (ZIKV; genus *Flavivirus*, family *Flaviviridae*) is a mosquito-borne virus that naturally cycles between vertebrate hosts and mosquito vectors. In urban environments, transmission predominantly occurs between humans and Aedes aegypti mosquitoes. In both field and laboratory settings, female A. aegypti mosquitoes have been shown to transmit ZIKV to their progeny at low rates, indicating that the virus can bypass the vertebrate host (1–4). Similarly, ZIKV can bypass the mosquito vector with direct human-to-human transmission. Of great clinical concern, vertical transmission during pregnancy can result in congenital Zika syndrome, a term for the combination of neuropathologic birth defects and disabilities following *in utero* exposure to ZIKV (5–8). ZIKV can also be transmitted horizontally in humans via sexual intercourse (9–15). Rare cases of horizontal transmission via blood transfusion (16), breastfeeding (17), and nonsexual contact (18) have also been reported. Rates of sexual ZIKV transmission and its contribution to epidemic spread are difficult to quantify in regions where A. aegypti is endemic, but the increased risk for seropositivity in sexual partners of index cases (19) and secondary cases in A. aegypti-free regions (8, 20) suggest it is a common non-vector-borne route of transmission. Given the aforementioned evidence for ZIKV mutations enhancing transmission and virulence, it is critical to study the impact of bypassing the mosquito vector on ZIKV evolution and the potential for adaptation to vertebrate hosts.

For arthropod-borne viruses (arboviruses), it has been proposed that a fitness trade-off occurs during host alternation, where fitness gains in one host are counteracted by fitness losses in the opposing host (21). However, this has not been supported by *in vivo* infection studies generally (22–25). Release from host alternation does typically enable adaptation to the vertebrate or arthropod but not necessarily at the cost of lost fitness in the other host. We hypothesized that ZIKV would demonstrate a similar capacity for adaptation to a vertebrate host when bypassing the mosquito vector, an outcome with potentially significant implications during direct ZIKV transmission in humans. Here, we tested this hypothesis by assessing phenotypic and genotypic changes following serial passage in mice or mosquitoes, and during alternating passage between both, using a molecularly barcoded ZIKV strain previously validated for tracking genetic bottlenecks and selective pressures within mosquitoes and nonhuman primates (26, 27). We found that ZIKV rapidly acquires enhanced virulence with universal fatality in mice coincidental to selective sweeps involving a previously described virulence-enhancing mutation. We additionally show that ZIKV populations evolve convergently under relaxed purifying selection in vertebrate hosts, whereas stochasticity and purifying selection characterize ZIKV evolution in mosquitoes and during alternating transmission.

## RESULTS

### Serial mouse or mosquito passage and alternating passage titers.

**FIG 1:**
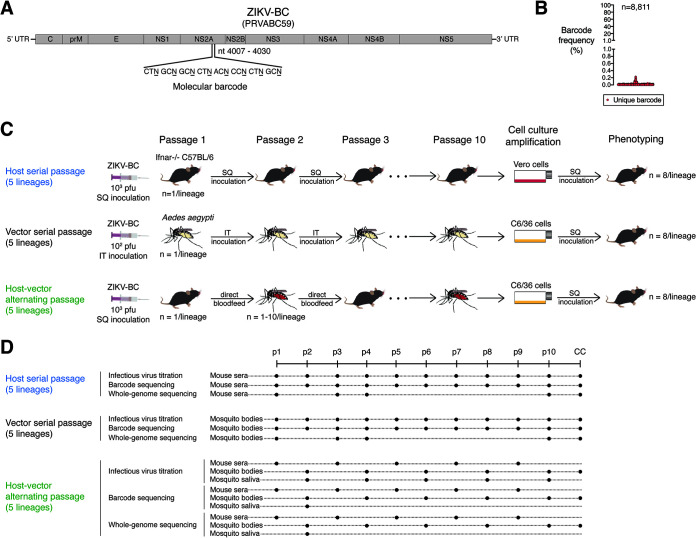
Genetic and phenotypic evolution of molecularly barcoded ZIKV tracked during serial or alternating *in vivo* passage. (A and B) Stocks of molecularly barcoded ZIKV (ZIKV-BC) consist of an unbiased distribution of unique barcodes. (C) Five replicate lineages of ZIKV-BC were serially passaged via needle inoculation for 10 passages in Ifnar1^−/−^ C57BL/6 mice or Aedes aegypti mosquitoes or passaged via bloodfeeding for 10 passages. Passage 10 viruses were amplified once in Vero or C6/36 cells before phenotypic analysis in mice. (D) Viral replication was tracked by plaque assay after each passage. Deep sequencing of virus barcodes and whole ZIKV genomes was employed to characterize virus population structure and composition, respectively, over the course of 10 passages. SQ, subcutaneous; IT, intrathoracic; CC, cell culture; UTR, untranslated region.

**FIG 2:**
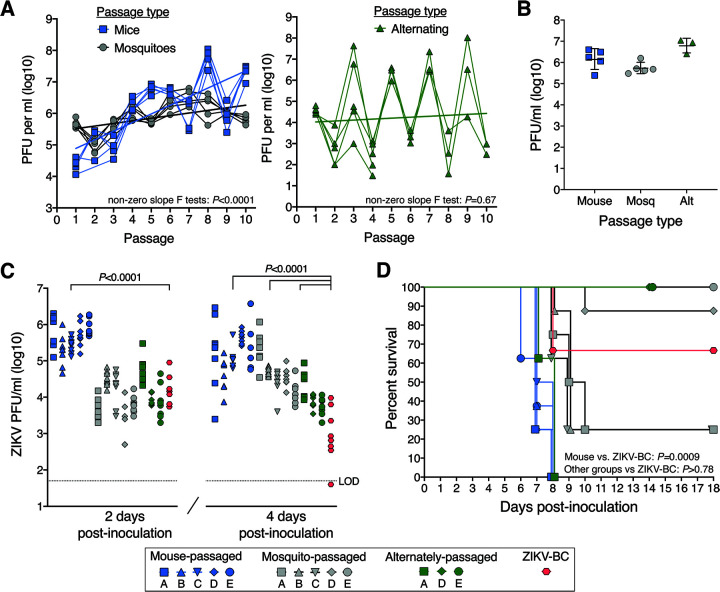
Phenotypic changes of serially and alternately passaged ZIKV-BC in mice. (A) Infectious ZIKV titers over sequential serial or alternating passages. (B) High titers of cell culture-amplified passage 10 viruses. (C) Infectious titers of passage 10 viruses and unpassaged ZIKV-BC stock in mouse sera 2 and 4 days postinoculation. *P* values shown are from ANOVA tests with multiple comparisons by Dunnett’s tests for each day postinoculation. (D) Survival of mice inoculated with passage 10 viruses and ZIKV-BC stock. *P* values shown are from Mantel-Cox log-rank tests between all replicate lineages of mouse-passaged (*n* = 40 mice), mosquito-passaged (*n* = 40 mice), or alternately passaged ZIKV (*n* = 24 mice) stock and the unpassaged ZIKV-BC stock (*n* = 8 mice). Alt, alternate; LOD, limit of detection. Symbol shapes and letters represent replicate lineages.

To determine the effect of release from host alternation on ZIKV evolution, *in vivo* serial passage experiments were conducted with a previously characterized barcoded ZIKV (ZIKV-BC; strain PRVABC59) containing a run of eight consecutive degenerate codons in NS2A (amino acids 144 to 151; Fig. 1A) that allows for every synonymous mutation to occur. There was no evidence of barcode bias in our ZIKV-BC stocks, as the 8,811 barcodes detected by deep sequencing were evenly distributed at less than 0.3% frequency within the population (Fig. 1B). ZIKV-BC was serially passaged in 5 parallel replicates (lineages) for 10 passages via subcutaneous (SQ) inoculation in *Ifnar1^−/−^* mice or via intrathoracic (IT) inoculation in A. aegypti mosquitoes (Fig. 1C). Despite a consistent amount of infectious virus inoculated at each passage, ZIKV-BC titers rose significantly over 10 serial passages in both mice and mosquitoes (*P < *0.0001 by non-zero slope F-test; Fig. 2A). The mean fold change in infectious virus titer from passage 1 to 10 was greater in mice (mean [standard deviation, or SD], +1,113 [636]) than in mosquitoes (+1.5 [0.4]).

To mimic natural host alternation, ZIKV-BC was alternately passaged in mice and mosquitoes for 10 passages in 5 parallel lineages. After SQ inoculation of mice on passage 1, alternating passage was conducted via natural bloodfeeding transmission, with small cohorts of mosquitoes feeding on an infected mouse and then feeding on a naive mouse 12 days later. Only 3 of 5 lineages successfully completed the series of 10 passages. Despite successful bloodfeeding, lineage B and C viruses replicated to very low titers (<10^2^ PFU/ml) in mosquito bodies on passage 4 and were not transmitted onwards to mice. Unlike with serial host and vector passage, infectious titers did not rise over the course of 10 alternating passages (*P = *0.67 by non-zero slope F test). The rising and falling sequential titers with alternating passage reflect the virus’ capacity for greater replication in mice than mosquitoes. After passaging ZIKV-BC with and without host alternation, we next assessed the passage 10 (p10) viruses for phenotypic changes in viral replication and virulence in mice.

### Phenotypic changes in mice.

Viral replication and virulence of mouse-passaged, mosquito-passaged, and alternately passaged p10 lineages were compared with unpassaged ZIKV-BC stock in *Ifnar1^−/−^* mice. To generate adequate viral stocks of the passaged viruses, each p10 virus isolate was amplified in a single passage at high multiplicity of infection on Vero cells for mouse-adapted lineages or on a mosquito cell line (C6/36) for mosquito-adapted and alternately passaged lineages (Fig. 2B). Cell culture-amplified stocks and p10 virus isolates were deep sequenced to ensure minimal changes in the single-nucleotide variant (SNV) frequencies after amplification (data not shown). To evaluate viral replication, infectious virus was titered by plaque assay at 2 and 4 days postinoculation (dpi) from mouse sera (Fig. 2C). At 2 dpi, only mouse-adapted ZIKV replicated to significantly higher titers than the unpassaged ZIKV-BC (*P < *0.0001 by one-way analysis of variance [ANOVA] with multiple comparisons by Dunnett’s test). By 4 dpi, mouse-adapted, mosquito-adapted, and alternately passaged lineages replicated to higher titers than the unpassaged ZIKV-BC (*P < *0.0001 by one-way ANOVA with multiple comparisons by Dunnett’s test). In terms of virulence, only the mouse-adapted lineages were associated with reduced survival in mice compared to unpassaged virus (*P = *0.0009 by Mantel-Cox log-rank test; Fig. 2D). Median survival was 7 dpi for mice infected with the mouse-adapted lineages, with all mice succumbing by 8 dpi. The mosquito-adapted and alternately passaged lineages demonstrated a wide range of virulence, with some lineages producing enhanced mortality rates relative to unpassaged virus and other lineages generating little to no mortality. These data demonstrate that host specialization can have various phenotypic effects on the direction of ZIKV virulence. Therefore, we next aimed to define genotypic diversity and the impact on ZIKV population structure/composition associated with differences in virus replication and virulence.

### Barcode dynamics.

We deep sequenced the barcode in virus populations from our passage series to track changes in the ZIKV population structure over sequential passages. For alternating passage 2, only mosquitoes with detectable infectious virus in their body and saliva were sequenced. In alternating passage lineages C and E, the dominant barcode in passage 3 and onwards was not detected in any of the sequenced mosquito tissues from passage 2, indicating that onward transmission was instigated by a mosquito whose saliva was not sequenced. As a result, mosquito bodies and saliva at passage 2 in lineages C and E were excluded from barcode analyses and figures. For alternating passages 4, 6, 8, and 10, barcodes only from mosquito bodies, and not saliva, were sequenced due to the minimal value added by sequencing populations dominated by a single barcode.

**FIG 3:**
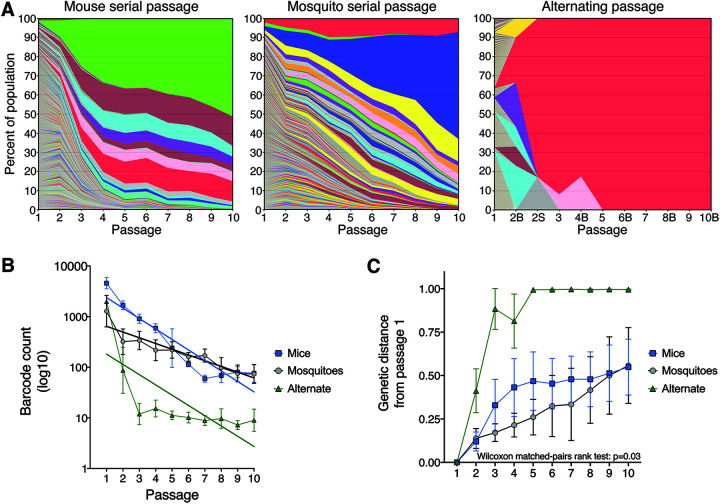
ZIKV barcode dynamics over sequential passages. (A) Individual barcode frequencies over 10 serial or alternating passages. Composite images for each passage series were generated by ranking barcodes from most to least frequent and calculating the mean frequency of the barcodes at each rank across the five replicate lineages. Colors represent barcode rank and are not associated with the barcode sequence. Thus, each colored bar is the mean frequency of barcodes with the same rank in the five replicate lineages. In the alternating passage series, even passages are labeled “B” or “S” to differentiate mosquito bodies and saliva, respectively. (B) Barcode abundance over sequential passages. Solid lines are linear regression lines of best fit. (C) Euclidean distance of barcode populations relative to populations at passage 1. Values ranging from 0 to 1 indicate degree of genetic similarity, with lower values indicative of high similarity and vice versa. Wilcoxon matched-pairs rank test result was reported for comparison between serial mouse and serial mosquito passage.

Across the five lineages serially passaged in mice, the virus populations were composed of more than 10^3^ uniquely barcoded viruses until passages 3 and 4, after which the populations were rapidly overtaken by a small number of barcoded viruses that remained dominant through passage 10 (Fig. 3A and B). In contrast, the virus populations serially passaged in mosquitoes exhibited a slower and steadier loss of population structure heterogeneity from approximately 10^3^ uniquely barcoded viruses to 10^2^ over the 10 passages (Fig. 3A and B). The divergence in the viral barcode populations relative to passage 1 was measured by Euclidean distance. Despite achieving comparable divergence by passage 10, the dynamics of genetic divergence in barcode populations differed significantly between serial mouse lineages and serial mosquito lineages over 10 passages (*P = *0.03 by Wilcoxon matched-pairs signed rank test; Fig. 3C). During serial mouse passage, rapid divergence in the first four passages was followed by slower divergence in the final six passages, whereas the rate of divergence was relatively stable during serial mosquito passage. For the alternately passaged populations, the population structure contracted down to only a few unique barcodes after the first passage in mosquitoes (Fig. 3A and B). In line with known anatomical bottlenecks in the mosquito midgut and salivary glands (28), constriction of the population structure was observed in mosquito bodies with further constriction in the mosquito saliva (Fig. 3A). Sudden homogenization of virus population structure, as seen during serial mouse and alternate passaging, is consistent with either a stringent genetic bottleneck or selective sweep(s).

**FIG 4:**
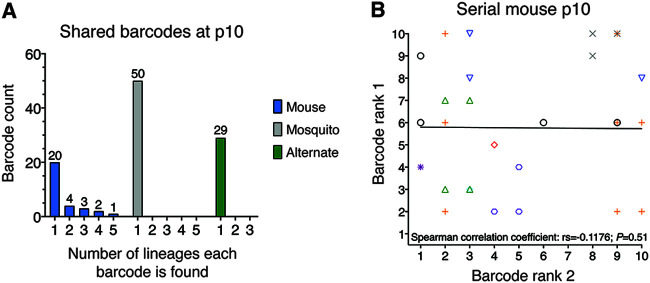
Barcodes shared between homotypic lineages at passage 10. (A) Histograms of the number of barcodes found in multiple (>1) lineages. Only the top 10 most frequent barcodes from each of the five lineages were analyzed (maximum of 50 barcodes). No barcodes were shared by multiple serial mosquito or alternating passage lineages, while 10 barcodes were shared by at least two serial mouse lineages. (B) Pairwise ranks for each of the 10 barcodes shared by more than one serial mouse lineage at passage 10. Colored symbols represent the 10 specific barcode sequences. The solid black line represents the line of best fit for all pairwise ranks. The nonparametric Spearman’s correlation coefficient (rs) and *P* value for all pairwise ranks are provided.

Importantly, none of the 50 most frequent barcodes present at passage 10 were shared between any mosquito lineage or alternate lineage (Fig. 4), indicating phenotypic neutrality for the barcodes *in vivo*. Of the 50 most frequent barcodes present at passage 10 in mouse lineages, 10 were present in more than one lineage, although there was no positive correlation between barcode frequencies across lineages (*P = *0.51 by Spearman’s correlation; Fig. 4). While selective advantages for certain barcodes in mouse cannot be ruled out, early selective sweeps and genetic hitchhiking can also cause barcode sharing if the barcode is linked with a nonbarcode mutation in the virus stocks that is selected for *in vivo*. Therefore, we conclude that the genetic barcodes are unbiased, neutral reporters of population evolution. In addition to evaluating ZIKV population structure, we also performed whole-genome deep sequencing to assess changes in the genetic composition of the ZIKV population.

### ZIKV adaptation to host associated with consistent emergence of NS2A A117V.

To capture the starting ZIKV populations, the period of greatest population change, and the final populations, we selected passages 1, 3, 4, and 10 for whole-genome deep sequencing (see Table S1 in the supplemental material). A targeted approach could not be applied to the alternating passage series, because the drastic decline in barcode abundance at passage 2 prevented the ascertainment of population changes after that point. Therefore, all 10 alternating passages were sequenced. The cell culture amplification passages for all passage series were also sequenced to confirm that virus amplification in cell culture had minor effects on the virus population. The frequency of individual SNVs was tracked over sequential passages to monitor the dynamics of ZIKV population composition. All SNVs called at greater than 1% frequency in any passage and with at least 300 reads of coverage were tracked. Depth of coverage was greater than 300 reads across the entire coding region for 81% (165/204) of sequencing libraries, with high coverage on at least 70% of the coding region in the remaining libraries. SNVs were further compared across lineages and passage series to assess convergent evolution. As with barcode sequencing, passage 2 from alternating passage lineages C and E was excluded from SNV analyses and figures, since the mosquito contributing to onward transmission was not sequenced. For the alternate passage lineages A, B, and D, passage 2 SNV data are from the single mosquito that contributed to onward transmission. SNV data from passages 4, 6, 8, and 10 for all alternate passage lineages are from mosquito pools, since the mosquito(es) contributing to onward transmission could no longer be identified by barcode sequences.

**FIG 5:**
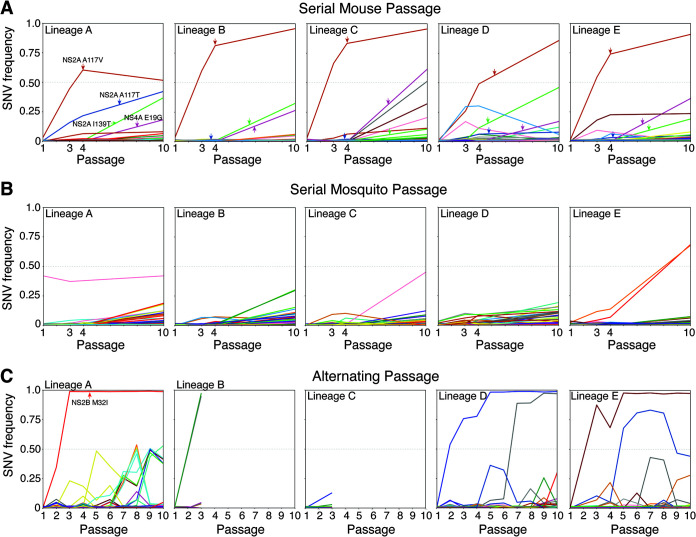
Trajectories of individual single-nucleotide variants (SNVs) over sequential passages detected at >1% frequency in any passage. (A to C) SNV frequencies for individual SNVs detected in serial mouse lineages (A), serial mosquito lineages (B), and alternating passage lineages (C). Four nonsynonymous SNVs detected in all five mouse lineages are demarcated with arrows, and one SNV of note is similarly demarcated in alternating passage lineage A. Colors represent the same SNVs across homotypic lineages but are used more than once due to the large number of SNVs. The same SNVs may not be represented by the same colors across passage types. In the alternating passage series, odd passages are mouse sera and even passages are mosquito bodies.

In the mouse serial passage lineages, four nonsynonymous SNVs, NS2A A117V, NS2A A117T, NS2A I139T, and NS4A E19G, arose in all five lineages, typically reaching high frequency in the population (Fig. 5A). Of particular note, the NS2A A117V rose from less than 2% frequency at passage 1 to greater than 25% by passage 3 and greater than 45% by passage 4. In four of the five lineages, NS2A A117V was present on more than 75% of viruses at passage 10. Interestingly, in lineage A, the frequency of NS2A A117V plateaued at just above 50%, but another mutation at the same locus, NS2A A117T, rose to 40% by passage 10, such that in this lineage viruses encoding alternate amino acids at NS2A residue 117 accounted for more than 90% of the population. The less frequent NS2A A117T mutation was also found at low frequency (<10%) in the other four lineages. The trajectory of the NS2A polymorphisms aligns closely with the aforementioned population structure dynamics (Fig. 3C), supporting our hypothesis that selective sweeps accounted for the dramatic loss in viral population diversity during mouse passage. The two other SNVs found in all five lineages, NS2A I139T and NS4A E19G, tended to arise between passages 4 and 10 and typically were found on less than 50% of the ZIKV genomes. None of the four presumed mouse-adaptive mutations demonstrated parallel trajectories indicative of genetic hitchhiking. The two NS2A 117 mutations and NS4A E19G were not detected in any passage of the serial mosquito and alternating passages. The NS2A I139T was detected in two serial mosquito lineages but never at greater than 2% frequency.

Unlike with serial mouse passage, we detected no SNVs shared by all 5 serial mosquito lineages above our 1% frequency cutoff (Fig. 5B). Across the five mosquito lineages, there were 204 unique SNVs that rose slowly over the 10 passages but remained below 25% frequency. Two SNVs in lineage E that exhibited parallel trajectories, indicative of genetic hitchhiking, were the only SNVs to achieve greater than 50% frequency. The observed SNV trajectory patterns in mosquitoes are consistent with “loose” transmission bottlenecks, where a high proportion of the inoculated virions infect and replicate, and weak positive selection following IT inoculation, such that SNVs arise, persist, and accumulate at lower frequencies. Similar to serial mosquito passage, alternating passage did not yield any high-frequency SNVs shared across the three lineages that progressed to passage 10 (Fig. 5C). One SNV, NS2B M32I, found at near 100% frequency in lineage A from passage 3 onwards, is a naturally occurring variant that was previously identified in pregnant rhesus macaques and associated with enhanced fetal infection in mice but reduced transmissibility in mosquitoes (referred to as M1404I) (29). To our knowledge, none of the other consensus-level SNVs detected in serial mosquito passage or alternating passage have been phenotyped. Unlike the accumulation of medium-frequency SNVs during serial mosquito passage, SNVs arising during alternating passage tended to either quickly rise to high frequency or be lost after a single passage. This SNV trajectory pattern in alternating passage lineages is consistent with sequential tight bottlenecks that drive SNVs to fixation or extinction.

### Differential selective pressures in mice and mosquitoes.

**FIG 6:**
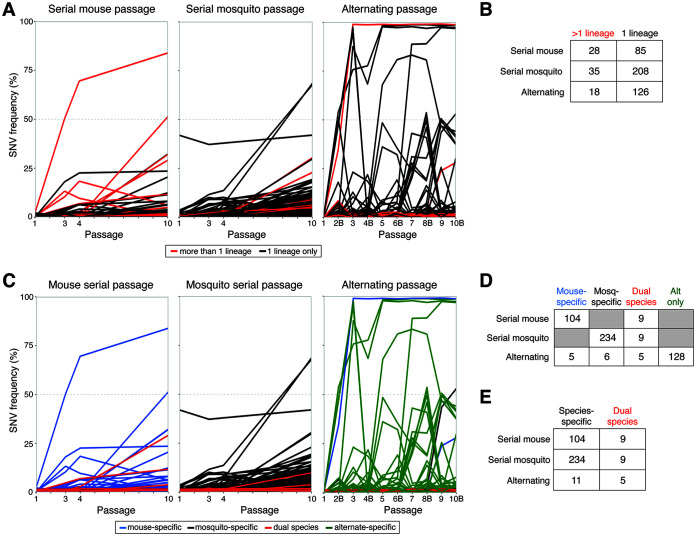
Convergence and species specificity of individual single-nucleotide variants (SNVs) detected at >1% frequency at any passage. (A) Convergent SNVs, colored red, were defined as being detected in more than one homotypic lineage at any passage number. (B) Abundance of convergent and nonconvergent SNVs for each passage series. (C) SNVs were defined as being mouse or mosquito specific if they were detected in one serial passage series and not the other. Dual-species SNVs were detected in both serial mouse and serial mosquito passage series. For the alternating passage series, any SNV not defined as species specific or dual species was defined as alternate specific. (D) Abundance of species-specific, dual species, and alternate-specific SNVs in each passage series. (E) Abundance of species-specific and dual-species SNVs in each passage series. For panels A and C, composite figures of SNV trajectories were generated by calculating the mean frequency of each SNV across all lineages it was detected in. For the alternating passage series, even passages are labeled “B” to indicate mosquito bodies.

In light of the shared SNVs arising during serial mouse passage, we sought to quantify the degree of convergent evolution in each passage type. Any variant (>1% frequency) that arose in more than one homotypic lineage was defined as convergent, and total convergence was quantified as the proportion of SNVs that were convergent (Fig. 6A and B). Across all the lineages, there was a greater degree of convergent evolution during serial mouse passage (25.4%) than serial mosquito (14.8%) or alternating passage (12.5%; X^2^, *P < *0.017). In the serial mosquito and alternating passage lineages, only 18.8% (6/32) and 6.5% (2/31) of high-frequency SNVs (>10% frequency), respectively, were shared across multiple homotypic lineages, whereas 53.8% (7/13) were shared across multiple serial mouse lineages.

In total, more than twice as many SNVs (243 versus 113) were detected in serial mosquito passage than serial mouse passage. Despite two alternating passage lineages ending at passage 4, more SNVs were also detected during alternating passage than serial mouse passage (144 versus 113). To understand the overlap in genetic sequence space explored by ZIKV during serial mouse or mosquito passage and alternating passage, we determined whether SNVs were species specific or found in both species. Each SNV detected during any passage at greater than 1% frequency was assigned as being mouse specific if it only arose during serial mouse passage and vice versa for mosquito-specific SNVs (Fig. 6C). Dual-species SNVs were those detected during both serial mouse and serial mosquito passage. For alternating passage, a fourth classification, alternate specific, was included for SNVs that only arose during alternating passage and never during serial mouse or mosquito passage. During serial mouse passage, 92.0% (104/113) of SNVs were mouse specific, whereas only 7.9% (9/113) were dual species (Fig. 6D). Similarly, during serial mosquito passage, 96.3% (234/243) were mosquito specific, whereas only 3.7% (9/243) were dual species (Fig. 6D). Taken together, these data indicate very little overlap in the sequence space explored by ZIKV during serial mouse and mosquito passage. Although more SNVs arose during serial mosquito passage, the proportion of species-specific SNVs was not significantly different than that of serial mouse passage (X^2^, *P = *0.09) (Fig. 6D). During alternating passage, 88.9% (128/144) of SNVs were alternate specific, while only 3.5% (5/144) were mouse specific, 4.2% (6/144) were mosquito specific, and 3.5% (5/144) were dual species. Again, these data demonstrate very little overlap in genetic sequence space used by species-adapted ZIKV and alternately passaged ZIKV. During alternating passage, mouse-specific SNVs were similarly likely to arise as mosquito-specific SNVs (*P = *0.28 by Fisher’s exact test; Fig. 6D), indicating no evolutionary advantage during alternating passage for SNVs associated with one species over the other species. In contrast, dual-species SNVs were significantly more likely to arise during alternating passage than species-specific SNVs (*P < *0.0001 by Fisher’s exact test; Fig. 6E), suggesting an evolutionary advantage during alternating passage for SNVs that arise during serial passage in both species.

**FIG 7:**
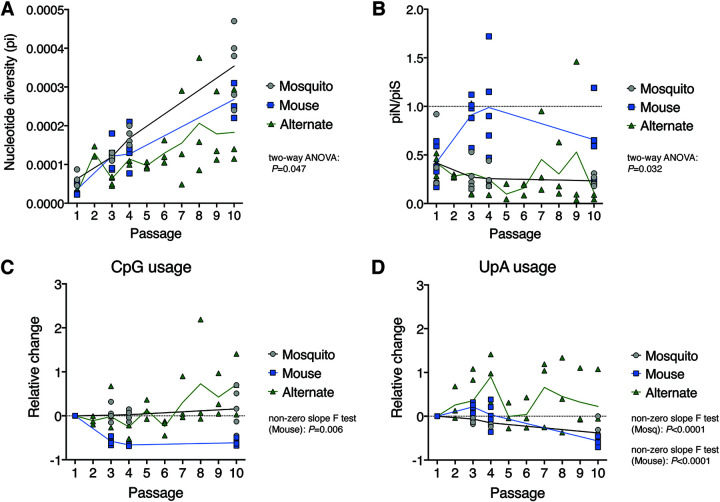
Genome-wide patterns of genetic diversity and dinucleotide usage. (A) Genome-wide nucleotide diversity (pi) in serial and alternate passage series over sequential passages. (B) Mean ratio of nucleotide diversity at nonsynonymous sites (piN) and synonymous sites (piS) across the genome. (C) Change in CpG usage relative to passage 1 in serial and alternate passage series. (D) Change in UpA usage relative to passage 1 in serial and alternate passage series. For panels A and B, two-way ANOVA *P* values are provided for comparison of all three groups at passages 1, 3, 4, and 10. For panels C and D, only statistically significant *P* values from linear regression nonzero slope F tests are provided. In all panels, solid lines represent mean values over sequential passages. Deep sequencing data were available for all 10 alternating passages but only passages 1, 3, 4, and 10 of the serial passage series. For serial passage series, *n* = 5 lineages at each passage. For alternating passage, *n* = 5 lineages at passages 1 to 3 and *n* = 3 lineages for passages 4 to 10. Even-numbered alternating passages are mosquito body samples. Alternate passage 2 data are from single mosquitoes that contributed to onward transmission, while alternate passage 4, 6, 8, and 10 are pools of all infected mosquitoes.

In addition to trends in individual SNVs, the relative effect of selective and stochastic mechanisms was compared between passage types using population-level metrics to better understand if host environments have different impacts on virus populations. Overall genome-wide population diversity, assessed by nucleotide diversity (pi), was comparable at passages 1 through 4 across all passage series (*P > *0.05 by two-way ANOVA with Tukey’s *post hoc* comparisons; Fig. 7A). By passage 10, serial mosquito lineages exhibited greater diversity than serial mouse lineages, which in turn were more diverse than alternating passage lineages (*P < *0.028 by two-way ANOVA with Tukey’s *post hoc* comparisons; Fig. 7A). The mutational spectra at passage 10 did not appear biased by technical artifacts, with transition substitutions occurring at a significantly greater frequency than transversion substitutions across all passage series (*P < *0.01 by matched one-way ANOVA with Dunnett’s *post hoc* comparisons; data not shown). The diversity at nonsynonymous versus synonymous sites (piN/piS) was employed as a proxy measurement for natural selection pressures, with values greater than 1 indicative of positive selection and less than 1 indicative of purifying selection. Mean piN/piS values were consistently less than 1 for all passage types but were significantly higher in serial mouse passages, suggesting that purifying selection was more relaxed in mice (*P = *0.032 by two-way ANOVA; Fig. 7B). Lastly, adaptation to host dinucleotide usage biases was compared between passage types by tracking changes in CpG and UpA dinucleotide usage within virus populations. In mice and humans, CpG and UpA dinucleotides are suppressed within the host genome (30), whereas only UpA dinucleotides are suppressed within mosquito genomes. In line with host and vector biases, CpG usage was strongly suppressed after the first serial passage in mice (*P = *0.0060 by linear regression non-zero slope F test), but no suppression was observed during serial mosquito or alternate passage (linear regression slope, >0; Fig. 7C). Similarly in agreement with host and vector biases, UpA usage was suppressed over serial passages in both mice and mosquitoes (*P < *0.0001 by linear regression non-zero slope F tests) but not over alternating passages (linear regression slope, >0; Fig. 7D).

## DISCUSSION

When cycling between vertebrates and mosquitoes, mosquito-borne viruses must alternately navigate distinct host environments and barriers to infection and transmission that restrict virus evolution. When cycling in humans, ZIKV can bypass mosquitoes via direct human-to-human transmission from mother to fetus or between sexual partners. It is unknown whether direct ZIKV transmission also occurs in vertebrate hosts of enzootic cycles, where ZIKV primarily evolved until recent epidemics. Infants infected *in utero* are likely dead-end hosts who do not contribute to onward transmission, but people infected by sexual transmission develop systemic infections and may transmit onwards to mosquitoes or additional sexual partners. Thus, direct human-to-human sexual transmission potentially enables ZIKV to redirect its evolutionary trajectory and quickly adapt to humans. In the current study, we model direct and alternating ZIKV transmission chains in mosquitoes and mice to elucidate the evolutionary pressures at play and the potential for adaptation to vertebrate hosts under different transmission conditions. We show that directly transmitted ZIKV rapidly adapts to mice, resulting in faster and higher rates of mortality. The rise in virulence repeatedly occurs in concert with the acquisition of one or two viral mutations of a single amino acid, NS2A A117V or A117T. In contrast, ZIKV virulence does not increase during natural host alternation by bloodfeeding transmission, and the NS2A mutations are never detected. Gains in replication and virulence following *in vivo* serial vertebrate passage have been observed with other mosquito-borne viruses (22, 24, 25, 31, 32), but convergent genetic evolution underlying the phenotypic changes has not been previously demonstrated *in vivo*.

In two strains of American-sublineage ZIKV not used here, NS2A A117V was previously shown to confer enhanced virulence in mice (33), but the phenotypic effect of NS2A A117T has yet to be determined and warrants further investigation. NS2A V117 is present on naturally isolated ZIKV genomes from humans and A. aegypti on the Virus Pathogen Resource database (ViPR; http://www.viprbrc.org), indicating it is a viable variant in natural transmission cycles. The NS2A T117 variant has not been previously detected in natural or laboratory isolates to our knowledge. In our study, the consistent emergence of the NS2A 117 substitutions coincidental with the sudden loss of barcode diversity is evidence for selective sweeps, as opposed to genetic bottlenecks where population diversity is lost but substitutions emerge randomly. Barcode sharing between serial mouse lineages after the selective sweeps indicates that the polymorphisms were present at below our 1% frequency cutoff in the ZIKV-BC stock and did not always arise *de novo* in mice. Interestingly, neither NS2A 117 substitution was detected in any serial mosquito or alternating passage. Two other uncharacterized substitutions, NS2A I139T and NS4A E19G, consistently arose during serial mouse passage, with only the former detected at low frequency in two serial mosquito lineages. The repeated emergence of these two substitutions, without aligned trajectories indicative of genetic hitchhiking, suggests they confer fitness advantages, but confirmatory phenotypic analyses are warranted. Neither mutation is present on any naturally isolated ZIKV genome in the ViPR database (http://www.viprbrc.org; accessed 28 December 2020). These findings indicate that restriction of ZIKV adaptation to vertebrate hosts during natural host alternation is robust, with nearly complete suppression of known and presumptive beneficial mutations consistently selected for when host alternation is circumvented. Thus, it is possible that releasing ZIKV from host alternation through direct human-to-human transmission may reduce the barrier for emergence of virulence-enhancing mutations.

Genetic drift and strong purifying selection appeared to be the predominant evolutionary forces during serial mosquito and alternating passage. This is supported by the paucity of convergent SNVs and lower genetic diversity at nonsynonymous sites than synonymous sites across the ZIKV genome. In contrast, there is evidence for directional selection and weak purifying selection during serial mouse passage, with a greater proportion of convergent SNVs and near equal diversity at nonsynonymous and synonymous sites. Stronger purifying selection during mosquito infection than during vertebrate infection has also been reported with chikungunya virus (genus *Alphavirus*, family *Togaviridae*) (34, 35) and dengue virus (genus *Flavivirus*, family *Flaviviridae*) (36–38), but opposite trends were observed with West Nile virus (genus *Flavivirus*, family *Flaviviridae*) with stronger purifying selection in vertebrates (25, 39, 40). The biological basis for the variability in within-host selective pressures acting on different arboviruses and its impact on virus evolution are unknown. The stochastic ZIKV evolution observed during alternating passage is, in large part, the result of tight genetic bottlenecks in mosquitoes during peroral infection and salivary transmission. Here, and in our previous studies (26, 27), barcoded ZIKV clearly highlights these bottlenecks via sequential, drastic reductions in barcode abundance in mosquito bodies and saliva. Furthermore, individual SNV trajectories during alternating passage display strong founder biases with SNVs either rising to dominance or being lost after mosquito infection. Bottleneck effects are not evident in ZIKV evolution during serial mosquito passage due the use of IT inoculation that bypasses the bottleneck sites in the midgut and salivary glands. IT inoculation was employed to model vertical transmission in mosquitoes and was additionally necessitated by the improbability of orally infecting naive mosquitoes with infectious, low-titer mosquito saliva.

Despite the preponderance of evidence for genetic drift and purifying selection during serial mosquito passage, we demonstrate that directional selection acts on dinucleotide usage, with UpA, but not CpG, dinucleotide usage being suppressed in mosquitoes. In serial mouse passage, we observed similar suppression of UpA dinucleotides but saw even more stringent suppression of CpG dinucleotides. The observed ZIKV dinucleotide usage patterns in mice and mosquitoes align with the dinucleotide usage biases in each host (30, 41). Mosquitoes and vertebrates exhibit disparate dinucleotide usage biases in their transcriptomes, and arbovirus genomes typically adopt an intermediate usage pattern at consensus level, unlike single-host viruses, presumably to accommodate both vector and vertebrate host environments (30, 42). To our knowledge, this is the first evidence of the dinucleotide selective pressure acting on a multihost virus at the subconsensus level as opposed to the consensus level. Unsurprisingly, we observed no clear trends in dinucleotide usage patterns during natural alternating passage, almost certainly the by-product of bottleneck events. This provides further evidence that natural alternating transmission restricts ZIKV’s capacity to adapt to vertebrate hosts and mosquito vectors.

To explain how alternating transmission restricts adaptation to hosts, the fitness trade-off hypothesis posits that fitness gains in one host come at the cost of fitness losses in the other host. Here, we demonstrate evidence to the contrary. Serial passage of ZIKV in mosquitoes increased viral replication in mosquitoes, but replication was not reduced in mice. These data are more consistent with the notion that the degree of host specialization can dramatically alter the evolution of virulence in pathogen populations and that a fitness gain in one environment may paradoxically broaden the overall phenotypic potential of a virus. To this end, we are performing additional studies to evaluate the phenotypic effects of host alternation release in mosquitoes to determine if the lack of fitness trade-offs is observed in both hosts. Antagonistic pleiotropy, i.e., contradictory phenotypes for the same mutations in hosts and vectors, is the rationale underlying the fitness trade-off hypothesis. Interestingly, although our data refute fitness trade-offs for host-adapted lineages, there is evidence for antagonistic pleiotropy, in that very few SNVs arise during both mouse and mosquito adaptation and very few species-specific SNVs arise during alternating transmission. This suggests that antagonistic pleiotropy can exist without apparent fitness trade-offs for the ZIKV population, possibly due to weak antagonism and overlapping viable fitness landscapes (43, 44). That said, species-specific SNVs (potentially antagonistic) were less likely to arise in alternately passaged ZIKV than dual-species SNVs that presumably had neutral or beneficial fitness effects in both hosts. Overall, alternately passaged ZIKV exhibited very little overlap in SNV usage with the mouse- or mosquito-adapted ZIKV. Nonmutually exclusive explanations for the uniqueness of alternately passaged ZIKV are (i) genetic drift in broad, viable sequence space such that few mutations are shared by chance and (ii) that host alternation acts as a selection pressure pushing the ZIKV population into a unique region of sequence space. Further investigations into the relative contribution of each explanation are worthwhile, because the likelihood of host-adaptive SNVs emerging is likely higher in the first scenario than the second. Additionally, making that distinction would inform whether ZIKV’s evolutionary potential under natural host alternation conditions can be explored or predicted by experimental adaptation to animal models or mosquitoes.

Taken together, our findings clarify the effect of host alternation on ZIKV evolution and highlight the potential for rapid adaptation to vertebrate hosts with direct vertebrate transmission chains. Whether similar adaptation would be observed with direct human-to-human transmission remains unclear. A potential limitation of this study is that direct transmission by needle inoculation may imperfectly model sexual transmission dynamics. In particular, the testes and epididymis are immune-privileged tissues maintained at lower than core body temperature and, therefore, may affect ZIKV evolution differently than other tissue compartments. Therefore, further studies are needed to assess ZIKV adaptation to vertebrate hosts using animal models of sexual transmission. Nonetheless, our data suggest that the prevention of direct human transmission chains should be a public health priority to thwart the emergence of virulence-enhancing mutations.

## MATERIALS AND METHODS

### Cells and virus.

African green monkey cells (Vero; ATCC CCL-81) and human embryonic kidney cells (HEK293T; ATCC CRL-3216) were cultured in Dulbecco’s modified Eagle medium (DMEM; Gibco) supplemented with 10% fetal bovine serum (FBS; Cytiva HyClone), 2 mM l-glutamine, 1.5 g/liter sodium bicarbonate, 100 U/ml penicillin, and 100 μg/ml streptomycin at 37°C in 5% CO_2_. Larval Aedes albopictus cells (C6/36; ATCC CRL-1660) were cultured in DMEM supplemented with 10% FBS, 2 mM l-glutamine, 1.5 g/liter sodium bicarbonate, 100 U/ml penicillin, and 100 μg/ml streptomycin at 28°C in 5% CO_2_. The barcoded ZIKV infectious clone was constructed by bacterium-free cloning of the ZIKV PRVABC59 strain genome (GenBank accession no. KU501215.1), as previously described (26, 27). Briefly, the ZIKV PRVABC59 isolate was passaged three times on Vero cells and twice on C6/36 cells, followed by PCR amplification of the whole genome in two overlapping amplicons. The genetic barcode, with degenerate nucleotides at the third position of 8 consecutive codons in *NS2A* (Fig. 1A), was then introduced via an overlapping PCR-amplified oligonucleotide. The two amplicons were assembled with a 5′ cytomegalovirus (CMV) promoter amplified from pcDNA3.1 (Invitrogen) by Gibson assembly (New England Biosciences [NEB]), followed by enzymatic digestion of the remaining single-stranded DNA and noncircular double-stranded DNA. Full-length ZIKV constructs were amplified using rolling circle amplification (repli-g minikit; Qiagen) and genomic integrity verified by restriction digestion and Sanger sequencing. Infectious barcoded ZIKV (ZIKV-BC) rescue was performed in HEK293T cells.

### Virus titration.

Infectious virus was titrated by plaque assay on Vero cells. A confluent monolayer of Vero cells was inoculated with a 10-fold dilution series of each sample in duplicate. Inoculated cells were incubated for 1 h at 37°C and then overlaid with a 1:1 mixture of 1.2% Oxoid agar and 2× DMEM (Gibco) with 10% (vol/vol) FBS and 2% (vol/vol) penicillin-streptomycin. After 4 days, the cell monolayers were stained with 0.33% neutral red (Gibco). Cells were incubated overnight at 37°C, and plaques were counted. Plaque counts were averaged across the two replicates, and the concentration of infectious ZIKV was back-calculated from the mean.

Viral RNA was isolated directly from mouse serum, mosquito saliva collected in cell culture media, and cell culture supernatant. Mosquito bodies were homogenized in phosphate-buffered saline (PBS) supplemented with 20% FBS and 2% penicillin-streptomycin with 5-mm stainless steel beads with a TissueLyser (Qiagen) prior to RNA isolation. RNA was isolated with the Maxwell RSC viral total nucleic acid purification kit on a Maxwell RSC 48 instrument (Promega). Isolated ZIKV RNA was titrated by qRT-PCR using TaqMan Fast virus 1-step master mix (ThermoFisher) and a LightCycler 480 or LC96 instrument (Roche). Final reaction mixtures contained 600 nM each ZIKV-specific qRT-PCR primer (5′-CGY TGC CCA ACA CAA GG-3′ and 5′-CCA CYA AYG TTC TTT TGC ABA CAT-3′) and 100 nM probe (5′-6-carboxyfluorescein-AGC CTA CCT TGA YAA GCA RTC AGA CAC YCA A-black hole quencher 1-3′) (45). Cycling conditions were 50°C for 5 min, 95°C for 20 s, and 50 cycles of 95°C for 15 s followed by 60°C for 1 min. ZIKV RNA titers were interpolated from a standard curve of diluted *in vitro*-transcribed ZIKV RNA. The limit of detection for this assay is 100 ZIKV genome copies/ml.

### Mice and mosquitoes.

*Ifnar1^−/−^* mice on the C57BL/6 background were bred in the specific-pathogen-free animal facilities of the University of Wisconsin-Madison (UW) Mouse Breeding Core within the School of Medicine and Public Health or in the specific-pathogen-free animal facilities of the University of Minnesota (UMN) College of Veterinary Medicine. Three- to 6-week-old mice of mixed sex were used for all experiments.

A. aegypti mosquitoes used in this study were maintained at UW and UMN using previously described rearing protocols (46). The A. aegypti line used in this study was established from several hundred eggs collected from ovitraps placed around the municipality of Buenos Aires (communa no. 9), a southeast suburb of Medellin, Colombia. Mosquitoes used in this study were from generations 3 to 30 of the laboratory colony. Three- to six-day-old female mosquitoes were used for all experiments.

This study was approved by the UW and UMN Institutional Animal Care and Use Committees (Animal Care and Use Protocol Numbers V5519 [UW] and 1804–35828 [UMN]).

### Serial passage in mice or mosquitoes.

Five *Ifnar1^−/−^* mice were subcutaneously inoculated in the left hind footpad with 10^3^ PFU of ZIKV-BC stock as passage 1 of five replicate lineages. Submandibular blood draws were performed 2 days postinoculation (dpi), and serum was processed for virus titration, sequencing, and onward passaging. Serial passaging for each lineage was maintained by inoculating a naive mouse with the 2-dpi serum diluted to 10^3^ PFU for 10 total passages. An inoculum titer of 10^3^ PFU for mouse passage was selected because it is biologically relevant for ZIKV titers in mouse ejaculate (47).

Female A. aegypti mosquitoes were anesthetized on ice and intrathoracically inoculated (48) with 100 PFU of ZIKV-BC in 1 μl. Inoculated mosquitoes were maintained on 0.3 M sucrose in an environmental chamber at 26.5°C ± 1°C, 75% ± 5% relative humidity, with a 12-h photoperiod within the Department of Pathobiological Sciences biosafety level 3 (BSL3) insectary at UW. At 12 dpi, mosquitoes were individually homogenized in 1 ml of PBS supplemented with 20% FBS and 2% penicillin-streptomycin. The supernatant was then collected and used for virus titration, sequencing, and onward passaging. Supernatant from five individual mosquitoes was then used to serially passage 100 PFU of virus through five replicate lineages of mosquitoes for 10 passages. An inoculum titer of 100 PFU for mosquito passage was selected because it approximates ZIKV titers in A. aegypti expectorant (49).

### Alternating passage.

Five *Ifnar1^−/−^* mice were subcutaneously inoculated in the left hind footpad with 10^3^ PFU of ZIKV-BC. Two days postinoculation, mice under ketamine-xylazine anesthesia were fed on by cartons of female A. aegypti that had been sucrose starved for 14 to 16 h prior to mouse feeding. After mosquito bloodfeeding, submandibular blood draws were performed to collect serum for virus titration and sequencing. Mosquitoes were anesthetized on ice, and mosquitoes that fed to repletion were selected and placed in new cartons containing an oviposition cup. Bloodfed mosquitoes were maintained on 0.3 M sucrose in an environmental chamber at 26.5°C ± 1°C, 75% ± 5% relative humidity, with a 12-h photoperiod within the Veterinary Isolation Facility BSL3 insectary at UMN. Twelve days postfeeding and following oviposition between 8 and 10 days, mosquitoes were bloodfed on new *Ifnar1^−/−^* mice. Mosquitoes were then triethylamine anesthetized, and saliva and whole bodies were collected from those that fed to repletion for sequencing and virus titration. Two days after bloodfeeding, these mice were fed on by naive cartons of mosquitoes to continue alternate passaging through 10 passages.

### Library preparation and sequencing.

Virus barcode libraries for the mouse and mosquito serial passage samples were generated with unique molecular identifiers (UMI) to filter sequencer and PCR errors that could produce false barcode sequences. ZIKV RNA concentrations in the alternating passage samples were too low to employ the UMI approach, so viral barcodes were sequenced by the whole-genome sequencing (WGS) approach described below. UMIs consisted of 12 random nucleotides inserted into the reverse primer used for reverse transcription of the ZIKV barcode region (5′-GGA GTT CAG ACG TGT GCT CTT CCG ATC TNN NNN NNN NNN NCC CCC GCA AGT AGC AAG GCC TG-3′). UMI-tagged cDNA was treated with RNase H, purified with magnetic beads (Agencourt RNAclean XP), and then PCR amplified for 20 cycles (forward, 5′-TCT TTC CCT ACA CGA CGC TCT TCC GAT CTT GGT TGG CAA TAC GAG CGA TGG TT-3′; reverse, 5′-GTG ACT GGA GTT CAG ACG TGT GCT CTT CC-3′; NEB Phusion master mix). Amplicons were bead purified (Agencourt Ampure XP) and then PCR amplified for 34 additional cycles using the same reverse primer and a forward primer bearing a 6-nucleotide index sequence (forward, 5′-CAA GCA GAA GAC GGC ATA CGA GAT NNN NNN GTG ACT GGA GTT CAG ACG TGT GCT CTT-3′). Reconditioning PCR using a 1/10 volume of the unpurified index amplicon was performed for 3 cycles using the same reagents as the index PCR. The entire volume of reconditioned UMI barcode libraries was purified by gel extraction (Qiagen QIAquick gel extraction kit).

Whole-genome ZIKV sequencing libraries were generated with a previously described tiled PCR amplicon approach (50, 51). Briefly, 10^6.15^ ZIKV genome copies were converted to cDNA with Superscript IV reverse transcriptase and random hexamer primers (ThermoFisher). PCR amplification of the entire ZIKV coding region was then performed in two reactions with pools of nonoverlapping PCR primer sets. Technical duplicates were generated for each WGS and UMI barcode library. All libraries were quantified by a Qubit 3 fluorometer (ThermoFisher) and quality assessed by an Agilent Bioanalyzer prior to sequencing. UMI barcode libraries were sequenced with paired-end 250-bp reads on an Illumina MiSeq (Illumina MiSeq reagent kit, v2). WGS libraries were sequenced with paired-end 150-bp reads on an Illumina NovaSeq 6000 by the UW Biotechnology Center (Illumina NovaSeq 6000 S1 reagent kit, v1.5).

### Bioinformatic analyses.

For UMI barcode sequence data, a pipeline was generated to process raw Illumina reads, extract consensus UMI reads, and calculate unique barcode abundance and frequencies. Briefly, raw paired-end reads were adapter and quality trimmed (q35), merged, cropped, and then quality filtered based on average base quality. High-quality reads were then grouped by UMI sequences, and, for UMI groups with at least 3 reads, the consensus sequence was extracted. The 24-nucleotide barcode sequence was then extracted from all consensus sequences without ambiguous bases. Finally, the abundance and frequency of each unique barcode sequence were calculated. For mosquito samples in alternating passage lineages, concentrations of ZIKV RNA were too low to use the UMI barcode library approach, so instead WGS data were used. First, reads were adapter and quality trimmed, and then any paired-end reads with mismatched bases in their overlapping sequences were filtered prior to merging. High-quality merged reads were aligned to the ZIKV PRVABC59 reference sequence, and reads fully covering the barcode region were isolated. Barcode sequences were then extracted from the reads, and the abundance and frequency of unique barcodes was calculated. For both the UMI and WGS barcode approach, mean barcode abundance and frequency was calculated across technical duplicate libraries and used in subsequent analyses.

For WGS data, a pipeline was generated to process raw Illumina reads, align reads at a normalized depth, call variants, and calculate diversity and dinucleotide usage metrics. Briefly, raw paired-end reads were adapter trimmed, and then any paired-end reads with mismatched bases or less than a 50-bp overlap were filtered prior to merging. Next, merged reads were quality trimmed, and primer sequences from the tiled primer sets were trimmed from the ends of the high-quality merged reads. Reads were then aligned to the ZIKV PRVABC59 reference and normalized to a coverage depth of approximately 2,500. Consensus sequences were extracted and variants were called against both the reference and consensus sequences with LoFreq* (52). As with barcode frequencies, variant frequencies were averaged across technical duplicate libraries, and mean frequencies were used for data analyses. Genome-wide and site-specific nucleotide diversities (pi, piN, and piS) were calculated with SNPGenie (v3; minfreq = 0.003) (53). Dinucleotide usage was calculated as the net change in frequency of each dinucleotide with a bespoke R script. First, dinucleotide sites were defined for each nucleotide pair across the reference genome, and then potential dinucleotide sites were identified as nucleotide pairs that differ from the target dinucleotide by one nucleotide (for example, CpC or ApG for CpG dinucleotides). The per-site dinucleotide losses then were calculated as the mean frequency of point mutations at each dinucleotide site, and per-site dinucleotide gains were calculated as the mean frequency of point mutations that generated the target dinucleotide at potential dinucleotide sites. Net dinucleotide usage for each dinucleotide was calculated as the per-site dinucleotide gains divided by per-site dinucleotide losses.

All bespoke data processing, analysis, and visualization scripts are publicly available on GitHub (https://github.com/tcflab/ZIKVBC_HostCycling). Read quality-trimming and cropping were conducted with Trimmomatic (v0.39) (54), cutadapt (v2.3) (55), and fastp (v0.20.0) (56). Read merging, alignment normalization, and barcode counting were performed with BBTools (v34.48; Joint Genome Institute). Reference alignment of reads was completed with the Burrows-Wheeler Aligner (bwa-mem; v0.7.16) (57). Parameter settings for each process not included in the text are provided in the aforementioned scripts.

### Statistical analyses.

All statistical analyses were conducted using GraphPad Prism 8 (GraphPad Software, CA, USA). Statistical significance was designated to *P* values of less than 0.05.

### Data availability.

Raw Illumina sequencing data are available on the NCBI Sequence Read Archive under BioProject no. PRJNA671510.
