# Correction for Riemersma et al., “Rapid Evolution of Enhanced Zika Virus Virulence during Direct Vertebrate Transmission Chains”

**DOI:** 10.1128/jvi.00501-22

**Published:** 2022-05-02

**Authors:** Kasen K. Riemersma, Anna S. Jaeger, Chelsea M. Crooks, Katarina M. Braun, James Weger-Lucarelli, Gregory D. Ebel, Thomas C. Friedrich, Matthew T. Aliota

**Affiliations:** a University of Wisconsin–Madison, Madison, Wisconsin, USA; b University of Minnesota, Twin Cities, St. Paul, Minnesota, USA; c Virginia Tech, Blacksburg, Virginia, USA; d Colorado State University, Fort Collins, Colorado, USA

## AUTHOR CORRECTION

Volume 95, no. 8, e02218-20, 2021, https://doi.org/10.1128/JVI.02218-20. Page 3, line 2 from the bottom: “*P = *0.0009” should read “*P = *0.0001.”

Page 4: In Fig. 2D, the ZIKV-BC trace was graphed incorrectly, indicating a larger effect than there actually is. The ZIKV-BC trace has been corrected to represent the correct values, and the *P* values have been adjusted.

Fig. 2D should appear as shown below.

**FIG 2:**
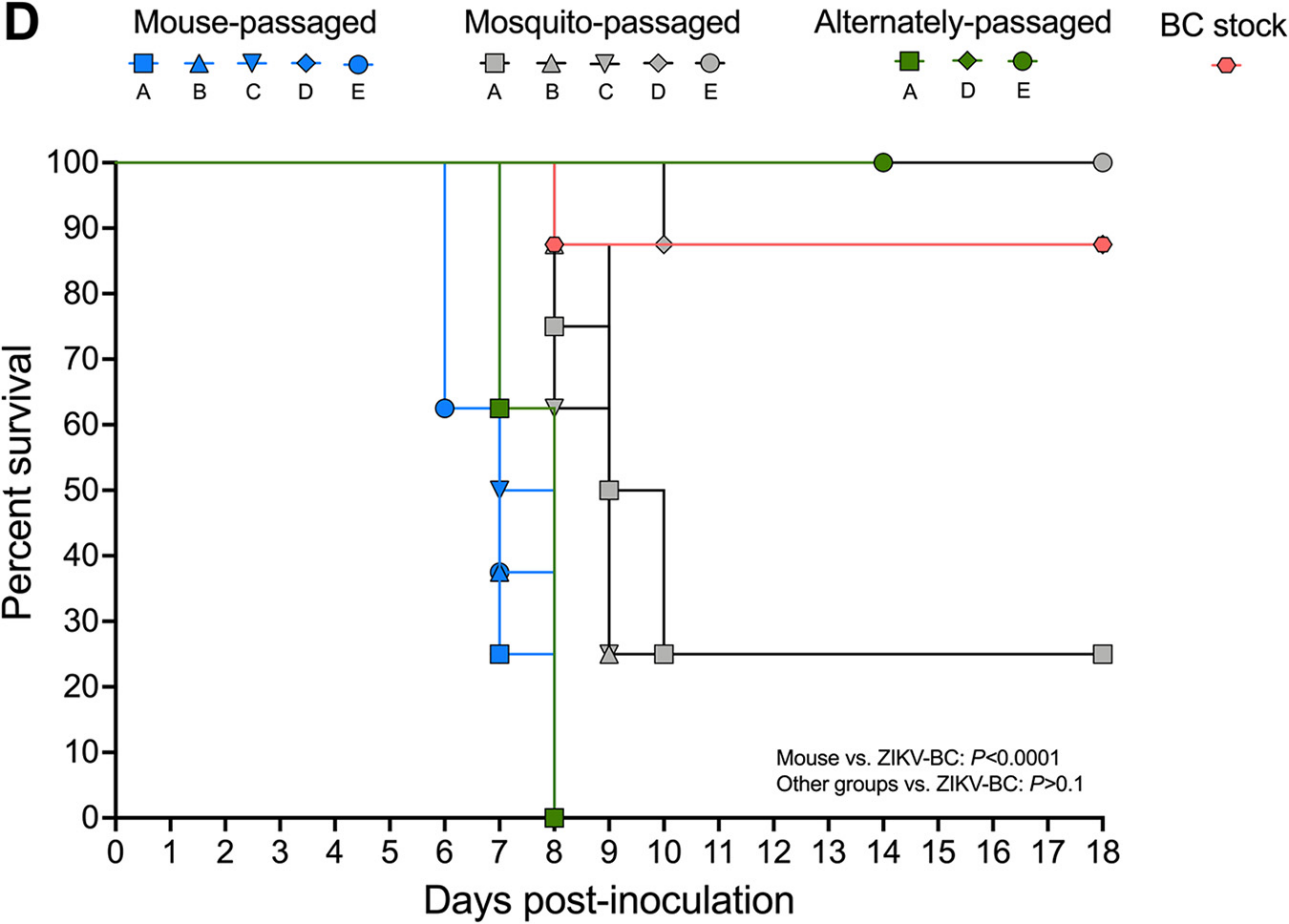


These errors do not alter the overall results or conclusions of the published article.

